# Thoracic radiotherapy and concurrent almonertinib for unresectable stage III EGFR-mutated non-small-cell lung cancer: a phase 2 study

**DOI:** 10.1186/s12885-021-08266-w

**Published:** 2021-05-07

**Authors:** Lucheng ZHU, Changlin Zou, Zhanchun Zhang, Jianfang Wang, Li Yang, Chuangzhou Rao, Zhiping Yang, Jiafeng Liang, Bing Xia, M. A. Shenglin

**Affiliations:** 1 Department of Thoracic Oncology, Key Laboratory of Clinical Cancer Pharmacology and Toxicology Research of Zhejiang Province, Affiliated Hangzhou Cancer Hospital, Zhejiang University School of Medicine, Hangzhou, 310002 People’s Republic of China; 2 Department of Radiotherapy, The First Affiliated Hospital of Wenzhou Medical University, Wenzhou, 325100 People’s Republic of China; 3 Department of Radiotherapy, Ningbo Medical Center Lihuili Hospital, Ningbo, 315100 People’s Republic of China; 4 Department of Radiotherapy, Shaoxing People’s Hospital (Shaoxing Hospital, Zhejiang University School of Medicine), Shaoxing, 312000 People’s Republic of China; 5 Department of Pulmonary & Critical Care Medicine, Shulan (Hangzhou) Hospital, Affiliated to Zhejiang Shuren University Shulan International Medical college, Hangzhou, 310022 People’s Republic of China; 6 Department of Radiotherapy & Chemotherapy,Hwa Mei Hospital, University of Chinese Academy of Sciences, Ningbo, 315010 People’s Republic of China; 7 Department of Oncology, Affiliated Hospital of Jiaxing University, The First Hospital of Jiaxing, Jiaxing, 314000 People’s Republic of China; 8 Department of Oncology, Jiande Second People’s Hospital, Hangzhou, 311604 People’s Republic of China; 9 Department of Cancer Medical Center, Affiliated Xiaoshan Hospital, Hangzhou Normal University, Hangzhou, 311201 People’s Republic of China

**Keywords:** Almonertinib, EGFR-TKI, Radiotherapy, Locally advanced NSCLC, Radiation pneumonitis

## Abstract

**Background:**

Concurrent chemo-radiotherapy remains the standard treatment in unresectable stage III non-small-cell lung cancer (NSCLC) patients. Several studies have shown a potential value of concurrent epidermal growth factor receptor-tyrosine kinase inhibitor (EGFR-TKI) with thoracic radiotherapy in EGFR-mutated population, but a high risk of radiation pneumonitis raised a major concern. This study intends to explore the safety and efficacy of concurrent almonertinib, a new third-generation EGFR-TKI, with radiotherapy in locally advanced EGFR-mutated NSCLC patients.

**Methods:**

Locally advanced NSCLC patients harboring sensitive EGFR mutation will be included in this study. A radiotherapy plan will be made for each patient before treatment, and the lung V20 will be calculated. Patients with lung V20 ≥ 28% were enrolled in induction group (arm A), which almonertinib was given for 2 months followed by concurrent radiotherapy; patients with lung V20 < 28% were enrolled in concurrent group (arm B), which almonertinib was given concurrent with thoracic radiotherapy. The primary endpoint is the incidence of grade ≥ 3 radiation pneumonitis within 6 months post-radiotherapy, and the secondary endpoints are local control rate, progression-free survival, and overall survival.

**Discussion:**

The safety and efficacy of third-generation EGFR-TKI concurrent with thoracic radiotherapy in locally advanced EGFR-mutated NSCLC is still unknown. We propose to conduct this phase 2 study evaluating the safety especially the radiation pneumonitis within 6 months post-radiotherapy. This trial protocol has been approved by the Ethics committee of Hangzhou cancer hospital. The ethics number is HZCH-2020-030.

**Trial registration:**

clinicaltrials.gov, NCT04636593. Registered 19 November 2020 - Retrospectively registered

## Background

Lung cancer is the leading cancer both worldwide and in China [1]. Non-small cell lung cancer (NSCLC) accounts for approximately 85% of all lung cancer cases and 30% of NSCLC patients are in stage III. Currently, the standard treatment for local advanced NSCLC is still doublet platinum-based therapy combined with radiotherapy, regardless of the EGFR mutation status. However, the treatment outcome is unsatisfied with an 5-year survival rate of less than 30% [2].

Many efforts including escalation of radiation dose, adjustment of chemotherapy regimen or sequence have been made to improve the outcome of local advanced NSCLC, but little progress was made. The PACIFIC study demonstrated maintenance immunotherapy with PD-L1 inhibitor durvalumab after chemoradiotherapy exerted large survival benefit, with median progression-free survival (PFS) of 16.8 months and 4-year overall survival of 49.6%, comparing to median PFS of 5.6 months and 4-year OS of 35.3% in the placebo group [3, 4]. However, subgroup analysis showed that patients with EGFR mutations might not benefit from maintenance immunotherapy.

For stage IV EGFR-mutant NSCLC, EGFR tyrosine kinase inhibitors (EGFR-TKIs) are the first-line treatment option. FLAURA study showed osimertinib prolonged mPFS to 18.9 months in treatment-naïve EGFR-mutant NSCLC patients. However, the value of EGFR-TKI in local advanced NSCLC is unknown. RECEL study was a randomized phase 2 trial comparing erlotinib and etoposide/cisplatin with concurrent radiotherapy for patients with stage IIIA/B unresectable advanced NSCLC with activating EGFR mutation. It showed the median PFS in first-generation EGFR-TKI combined with thoracic radiotherapy (24.5 months) was significantly improved compared with chemoradiotherapy (9.0 months) [5]. It suggests the potential value of concurrent EGFR-TKI with radiotherapy.

Another concern is that concurrent TKI and radiotherapy could increase the incidence of radiation pneumonitis. Previous studies showed that the incidence of radiation pneumonitis in combination radiotherapy with first-generation TKI was as high as 40%, and grade ≥ 3 pneumonitis rate reached 20% [6]. Severe radiation pneumonitis might counteract the benefit of EGFR-TKI and radiotherapy. Therefore, it is essential to explore the safety of TKI-radiotherapy combination therapy.

Almonertinib, HS-10296, is a third-generation EGFR-TKI developed by Hansoh pharmaceutical company in China. It can irreversibly bind to mutated EGFR, specifically to T790M, exon 21 L858R, and exon 19 deletion. At the 2020 AACR annual meeting, Lu et al. presented the preliminary results of APOLLO study, a phase 2 trial [7]. All patients progressing on prior EGFR-TKI treatment and harboring T790M mutation received almonertinib at a dose of 110 mg per day. The object response rate (ORR) and disease control rate (DCR) were 68.9 and 93.4%. The median PFS was 12.3 months. The incidence of grade 3 or higher adverse events (AEs ≥ 3) was 15.6%. The most common AEs were increased creatine phosphokinase, skin rash, increased aspartate aminotransferase, increased alanine aminotransferase et al. Notably, there was no interstitial lung disease reported. China Food and Drug Administration has therefore approved almonertinib for EGFR-sensitive-mutation or T790M mutation NSCLC patients progressing to prior EGFR-TKIs.

There are few knowledges about the safety of third generation EGFR-TKI combined with thoracic radiotherapy. This study aims to explore the safety of concurrent radiotherapy with almonertinib in EGFR-mutant local advanced NSCLC patients, most importantly the incidence of radiation pneumonitis.

## Methods and design

### Study design

**Fig. 1:**
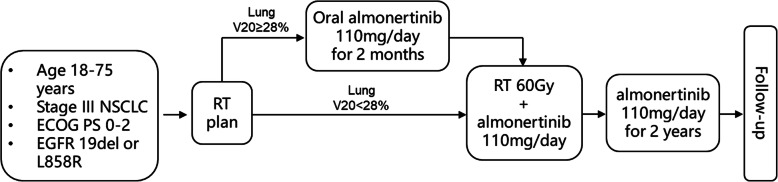
Scheme of the study

The study is designed as a multi-center phase 2 study to evaluate the safety of concurrent radiotherapy with almonertinib in EGFR-mutated locally advanced NSCLC patients. Previous studies have demonstrated higher lung V20 correlated with higher the probability of radiation pneumonitis [8]. In order to reduce the influence of large PTV on radiation pneumonitis, patients with lung V20 ≥ 28% were enrolled in induction group (arm A), which almonertinib was given for 2 months followed by concurrent radiotherapy; patients with lung V20 < 28% were enrolled in concurrent group (arm B), which almonertinib was given concurrent with thoracic radiotherapy (Fig. 1).

### Primary endpoint

The incidence of radiation pneumonitis (≥ grade 3) within 6 months post-radiotherapy.

### Secondary endpoints

· Local control rate

· Progression-free survival

· Overall survival

### Inclusion criteria

· Histologically or cytologically confirmed NSCLC.

· Inoperable stage III according to the eighth edition of TNM classification system.

· Blood or tissue EGFR detection harboring exon 19 deletion or L858R mutation.

· Forced expiratory volume in 1 s > 0.75 L.

· Age ≥ 18 years old.

· Eastern Cooperative Oncology Group (ECOG) performance status (PS) ≤ 2.

· Estimated survival period ≥6 months.

· Signed the informed consent form.

### Exclusion criteria

· Patients who received prior anti-tumor treatments, including chemotherapy, immunotherapy or EGFR-TKIs.

· If lung V20 ≥ 28% even after 2-month induction almonertinib.

· Contraindications to radiotherapy.

· Other malignant tumors within 5 years (except for non-melanoma skin cancer and cervical cancer).

· Any medical or non-medical reasons prevent the patient from continuing to participate in the research.

· Use of any drugs or substances known to be strong or moderate inhibitors or inducers of CYP3A4 within 30 days prior to study.

· Pregnancy or breastfeeding.

### Treatment planning

All enrolled patients underwent positioning CT scans before the initial treatment, a radiotherapy plan was made for each patient, and the lung V20 of each patient was calculated. Patients with lung V20 ≥ 28% were enrolled in Arm A: Almonertinib was administered for 2 months followed by almonertinib combined with radiotherapy; patients with lung V20 < 28% were enrolled in Arm B: almonertinib was administered concurrent with radiotherapy. Almonertinib will be administered oral 110 mg/day for 2 years or until disease progression or intolerable toxicity.

### Radiotherapy

4D-CT with intravenous contrast should be used for all patients. Gross tumor volume (GTV) is defined as volume of the primary tumor mass and nodal diseases. Involved field radiotherapy is adopted in this study, while elective nodal irradiation is not permitted. The clinical target volume (CTV) will consist of the GTV and approximately a 0.5 cm margin for microscopic extension. The plan target volume (PTV) will consist of the CTV and approximately a 0.5 cm margin for patient movement and setup uncertainties.

Radiotherapy will be delivered at least intensity modulated radiotherapy (IMRT). Radiotherapy dose is set to 60Gy ± 10%. The definition target volume refers to ICRU50 and ICRU62.

Dose limits for normal tissues and organs are as follows: mean lung dose<20Gy and/or V20 < 28%; mean esophageal dose<34Gy; heart V50 < 25%, V30 ≤ 50% and V45 < 35%.

Cone-beam computed tomography (CBCT) or other position verification techniques are required to verify the accuracy of patient position every week.

Interruption of radiotherapy: Interruption of radiotherapy should be avoided as much as possible. The following situations can be suspended radiotherapy: 1) The absolute number of neutrophils is less than 1.0 × 10^9^/L; 2) Platelets are less than 50 × 10^9^/L; 3) ≥2 radiation pneumonitis; 4) Diarrhea (water samples per day Diarrhea ≥7 times), those who are ineffective after antidiarrheal treatment; 5) Weight loss ≥20%; 6) Any unexpected adverse effects that might or might not be related to the treatment should be determined by investigators.

Recovery of radiotherapy: Continue treatment until adverse effects improve to Grade ≤ 2, except that radiation pneumonitis should improve to Grade ≤ 1.

Termination of radiotherapy: 1) After active management, the adverse effects have not improved to Grade ≤ 2; 2) The interruption of radiotherapy due to any reason is more than 10 days; 3) The investigator considers that it is necessary to terminate radiotherapy; 4) The patient requests to terminate radiotherapy.

### Safety monitoring and tumor evaluation

All lesions must be recorded during the screening period and re-evaluated in each subsequent tumor evaluation. Tumor will be evaluated during screening, before drug administration and radiotherapy, and follow-up every 8 weeks after the start of the study.

Medical history, demographic data, complete physical examination, vital signs, ECOG score, and laboratory tests will be recorded at each follow-up. Adverse effects will be evaluated according to National Cancer Institute Common Terminology Criteria for Adverse Events version 5.0 (NCI CTCAE 5.0).

### Statistical analysis

A Simon’s Two-Stage design is used in this study. The null hypothesis of the trial is that the 6-month pneumonitis-free rate of grade ≥ 3, was less or equal to 75% versus alternative of at least 90%. In this study, *n* = 43 patients are needed for testing the null hypothesis at a one-sided significance level of α = 0.05 with a probability of 1–β = 0.8. At the first stage, if 5 of the 22 patients experienced grade ≥ 3 radiation pneumonitis, then the study is stopped for futility.

All data will be reviewed by an independent Data Monitoring Committee (IDMC).

## Discussion

The main purpose of this study is to explore the safety of third-generation EGFR-TKI almonertinib combined with radiotherapy for stage III NSCLC patients. The incidence of radiation pneumonitis is one of the most important indicators for this study.

Third-generation EGFR-TKI could increase radiosensitivity in EGFR-mutant NSCLC. Yu et al. showed third-generation EGFR-TKI could reduce G2/M-phase cell cycle arrest and block IR-induced DNA DSB repair [9]. Ma et al. found that third-generation EGFR-TKI increased sensitivity to radiation by delaying DNA damage repair after irradiation and inducing apoptosis by the EGFR signaling pathway [10]. However, previous studies have showed that EGFR-TKI combined with radiotherapy can also increase the incidence of radiation pneumonitis. The EGFR signaling pathway is the most widely studied growth factor receptor. It is universal in cells and has a variety of signal transduction functions. EGFR is widely distributed in alveolar epithelium, submucosal glands, bronchial epithelial basal layer and endothelium in the respiratory system. EGFR pathway plays a critical role in repairing airway damage, promoting epithelial cell migration and proliferation. When the lung tissue is injured by radiotherapy, the EGF signaling pathway can promote the proliferation of alveolar epithelial cells to repair the integrity of the alveolar wall, thereby repairing lung injury [11]. EGFR-TKI could interferes with the EGF signaling pathway and affect the repair of lung tissue. The study of Zheng et al. showed that the incidence of radiation pneumonitis in combination radiotherapy with first-generation TKI was as high as 40%, and grade ≥ 3 pneumonitis rate reached 20% [6]. RECEL study also showed grade ≥ 3 radiation pneumonitis was 16.7% in the EGFR-TKI concurrent group [12]. In fact, first-generation EGFR-TKI could not only inhibit mutant EGFR, but also potently inhibit wild-type EGFR. To the contrary, third-generation EGFR-TKI have low selectivity for wild-type EGFR, which might explain its low incidence of interstitial pneumonia [13].

Almonertinib is a third-generation EGFR-TKI. APOLLO study showed almonertinib had a favorable safety profile with no interstitial pneumonia occurred in the included patients. While the safety of almonertinib concurrent with thoracic radiotherapy is still unknown. Jia et al. reported an especially high rate of grade 2 or worse RP in patient treated with combination thoracic radiotherapy and osimertinib. Seven of 11 patients (63.6%) experienced grade 2 or worse radiation pneumonitis and 5 (45.4%) exhibited grade 3. Of course, Jia’s study was a retrospective study with small sample size and, bias might influence the results. Therefore, a well-designed prospective study is needed to explore the safety of third-generation EGFR-TKI combined with thoracic radiotherapy for locally advanced NSCLC patients.

## Data Availability

The datasets used and/or analyzed during the current study are available from the corresponding author on reasonable request.

## Abbreviations

AEs: Adverse events
CBCT: Cone-beam computed tomography
CTCAE: Common terminology criteria
CTV: Clinical target volume
DCR: Disease control rate
EGFR: Epidermal growth factor receptor
GTV: Gross tumor volume
ICRU: International commission on radiation units & measurements
IMRT: Intensity modulated radiotherapy
NCI: National cancer institute
NSCLC: Non-small cell lung cancer
ORR: Object response rate
OS: Overall survival
PFS: Progression-free survival
PTV: Plan target volume
TKI: Tyrosine kinase inhibitor

## References

[1] Chen W, Zheng R, Baade PD, Zhang S, Zeng H, Bray F, Jemal A, Yu XQ, He J. Cancer statistics in China, 2015. CA Cancer J Clin. 2016;66(2):115-32. 10.3322/caac.21338 26808342

[2] Howlader N, Noone AM, Krapcho M, Miller D, Brest A, Yu M, Ruhl J, Tatalovich Z, Mariotto A, Lewis DR, Chen HS, Feuer EJ, Cronin KA. SEER Cancer Statistics Review, 1975-2017. Bethesda: National Cancer Institute; 2020. https://seer.cancer.gov/csr/1975_2017/.

[3] Antonia SJ, Villegas A, Daniel D, Vicente D, Murakami S, Hui R, Kurata T, Chiappori A, Lee KH, de Wit M, Cho BC, Bourhaba M, Quantin X, Tokito T, Mekhail T, Planchard D, Kim YC, Karapetis CS, Hiret S, Ostoros G, Kubota K, Gray JE, Paz-Ares L, de Castro Carpeño J, Faivre-Finn C, Reck M, Vansteenkiste J, Spigel DR, Wadsworth C, Melillo G, Taboada M, Dennis PA, Özgüroğlu M (2018). Overall survival with Durvalumab after Chemoradiotherapy in stage III NSCLC. N Engl J Med.

[4] Faivre-Finn C, Vicente D, Kurata T, Planchard D, Paz-Ares L, Vansteenkiste J, Spigel D, Garassino M, Reck M, Senan S (2020). LBA49 Durvalumab after chemoradiotherapy in stage III NSCLC: 4-year survival update from the phase III PACIFIC trial. Ann Oncol.

[5] Xing L, Wu G, Wang L, Li J, Wang J, Yuan Z, et al. Erlotinib vs etoposide/cisplatin with radiotherapy in unresectable stage III epidermal growth factor receptor mutation-positive non-small-cell lung cancer: a multicenter, randomized, open-label, phase 2 trial. Int J Radiat Oncol Biol Phys. 2020;109(5):1349-58. 10.1016/j.ijrobp.2020.11.026. 10.1016/j.ijrobp.2020.11.026 33220395

[6] Zheng L, Wang Y, Xu Z, Yang Q, Zhu G, Liao XY, Chen X, Zhu B, Duan Y, Sun J (2019). Concurrent EGFR-TKI and thoracic radiotherapy as first-line treatment for stage IV non-small cell lung Cancer harboring EGFR active mutations. Oncologist.

[7] CT190 - A multicenter, open-label, single-arm, phase II study: The third generation EGFR tyrosine kinase inhibitor almonertinib for pretreated EGFR T790M-positive locally advanced or metastatic non-small cell lung cancer (APOLLO). In: American Association for Cancer Research: 2020; 2020.

[8] Hanania AN, Mainwaring W, Ghebre YT, Hanania NA, Ludwig M (2019). Radiation-induced lung injury: assessment and management. Chest.

[9] Wang N, Wang L, Meng X, Wang J, Zhu L, Liu C, Li S, Zheng L, Yang Z, Xing L, Yu J (2019). Osimertinib (AZD9291) increases radio-sensitivity in EGFR T790M non-small cell lung cancer. Oncol Rep.

[10] Wu S, Zhu L, Tu L, Chen S, Huang H, Zhang J, Ma S, Zhang S (2018). AZD9291 increases sensitivity to radiation in PC-9-IR cells by delaying DNA damage repair after irradiation and inducing apoptosis. Radiat Res.

[11] Aida S, Tamai S, Sekiguchi S, Shimizu N. Distribution of epidermal growth factor and epidermal growth factor receptor in human lung: immunohistochemical and immunoelectron-microscopic studies. Respiration. 1994;61(3):161-6. 10.1159/000196329 8047720

[12] Xing L, Wu G, Wang L, Li J, Wang J, Yuan Z, Chen M, Xu Y, Fu X, Zhu Z et al: Erlotinib vs etoposide/cisplatin with radiotherapy in unresectable stage III epidermal growth factor receptor mutation-positive non–small-cell lung cancer: A multicenter, randomized, open-label, phase 2 trial. Int J Rad Oncol Biol Phys 2020. 10.1016/j.ijrobp.2020.11.026 33220395

[13] Wang S, Cang S, Liu D (2016). Third-generation inhibitors targeting EGFR T790M mutation in advanced non-small cell lung cancer. J Hematol Oncol.

